# No consensus about consensus?

**DOI:** 10.1186/s42466-021-00144-x

**Published:** 2021-08-06

**Authors:** Ludwig Kappos

**Affiliations:** Research Center Clinical Neuroimmunology and Neuroscience Basel (RC2NB), University Hospital and University of Basel, Spitalstrasse 2, CH-4031 Basel, Switzerland

MS as a chronic, heterogeneous disease with a pathogenesis that derives from the complexities of both the immune and central nervous system lends its self to evidence based treatment guidelines and consensus statements to guide therapeutic decisions. This is even more the case in view of the rapidly increasing number of available compounds and therapeutic strategies that are now available for the different stages of the disease.

Guidelines and consensus statements aim to provide orientation to the less specialized health care professional, inform patients and help in defining common standards of care and harmonizing treatment policies, often with implications for reimbursement. With such a wide range of implications, including important financial interests, the value and acceptance of guidelines depends on the transparency of the development process and the scientific and professional authority and recognition of those involved, particularly when it comes to “softer” recommendations that are not derived from unequivocal evidence.

German neurologists, with the participation of authors from Austria and Switzerland, have a tradition of valuable contributions: Since the earlier recommendations by the MS Therapy Consensus Group (MSTKG) [1, 2] and more recently by the Competence Network Multiple Sclerosis (https://www.kompetenznetz-multiplesklerose.de/wp-content/uploads/2018/11/KKNMS_Qualit%C3%A4tshandbuch-MS-NMOSD_2018_webfrei.pdf) the authors have not refrained from providing consensus recommendations on clinically important topics where evidence was still scarce. With its 2021 Guideline [3] the German Neurological Society presented a comprehensive and thoroughly revised document. Although derived in adherence to the procedures defined by the Association of the Scientific Medical Societies in Germany for “S2k level consensus-based guidelines”,(https://www.awmf.org/leitlinien/awmf-regelwerk/ll-entwicklung/awmf-regelwerk-01-planung-und-organisation/po-stufenklassifikation/klassifikation-s2k-und-s2e.html) some of the statements were criticized by other leading MS experts – some involved in the GNS guideline committee [4]. As this debate is about key and timely issues of high interest in MS that deserve attention in a broader clinical scientific audience the editor of Neurological Research and Practice asked the protagonists of the debate to answer four questions that address the central topics of controversy [5, 6].

The authors of the two statements can be commended for their well thought out contributions. They explain the respective rationales e.g. interpretations of current evidence from clinical trials and “real life” studies underlying concepts of disease pathogenesis and mode of action of DMTs and allow the readers to draw their own conclusions.

My overall impression is that disagreement may be less than initially assumed.

*Question 1* is about early initiation of treatment: Both agree that prevention of disease activity (as depicted by MRI lesions and relapses) and worsening (as depicted by thorough and comprehensive assessment of CNS function) and not reaction to already established damage is the preferred strategy. There is also agreement that early treatment is the most effective strategy to reach these goals. *Differences exist regarding how early is early and the rigor of recommending treatment.* Bayas and colleagues [5] suggest an expectative attitude in CIS patients who do not fulfill the formal criteria for dissemination in space. They also seem more inclined to accept exceptions to the therapeutic imperative in established MS if “individual patient characteristics are indicative of a very favorable disease course” but also declare that the option not to initiate DMT “is clearly an exception, applicable only to a small proportion of patients”. Wiendl et al. [6] take the stand that all patients with established MS diagnosis, but also all CIS patients, need to be offered immunotherapy, regardless of whether the criteria for dissemination in time and space are met, as long as there is no other plausible differential diagnosis.

Taking into account that the definition of a benign disease course and its possible predictors remains elusive [7], and that in those controlled studies that have shown the positive benefit/risk balance of initiating treatment early in CIS participation was not restricted to fulfillment of the current criteria for DIS I am inclined to follow the approach proposed by Wiendl et al. in my daily practice.

*Question 2* is about the selection of DMT and treatment strategy: Again both agree that an individual assessment of disease characteristics and potential risks should guide the choice of treatment and that the full spectrum of available options needs to be considered even in very early stages of MS and in treatment naïve patients. When it comes to defining the candidates for higher efficacy treatments Bayas et al. [5] restrict this option to treatment failures of “category 1” compounds and – in treatment naïve patients - to “probably highly active” disease that they define following recommendations from a recent workshop about “aggressive” MS [8]. Wiendl et al. [6] suggest a more open approach to early high efficacy treatment, being encouraged by accumulating evidence from several registry based observational studies supporting the benefits of early high efficacy treatments [9, 10]. Both author groups acknowledge that results of controlled prospective studies comparing the benefit-risk balance of the early high efficacy treatment approach (“hit hard and early”) versus an escalation approach are still pending. Bayas et al. categorize available DMDs in three groups mainly according to their effects on relapse rates in different studies and propose an escalating approach for the majority of patients. Wiendl et al. underscore that each of the available treatments deserves a thorough appraisal for each individual with MS but still mention a number of DMDs that they classify as preferentially indicated in “mild to moderate” MS. *If an optimal control of disease activity and progression is our goal only concerns about risk and tolerability can prevent from using the most effective treatment options. *The manageable risk and convenience profile emerging from long term extensions of large controlled studies [11–13] and from systematic follow up in real world settings [9, 10] for some of the high efficacy treatments, namely S1P modulators, anti-CD20 monoclonal antibodies and natalizumab (in JC virus negative patients and probably even more with extended interval dosing) [14] alleviate such concerns more and more.

*Question 3* refers to long term continuation of DMTs. We all know that MS is still a disease without cure. With the exception of intensive cell depleting therapies that are given intermittently and may have long term effects thereafter - even leading to “immune reconstitution” and permanent recovery in a proportion of patients, all other treatments need to be given continuously to achieve their effects. Nevertheless, a substantial proportion of pwMS (up to 60–70% in different surveys) take only part of the prescribed medication or discontinue treatment within the first 1–5 years after initiation [15, 16]. Should neurologists sanction such practice in stable patients? Bayas et al. [5] - although stating that no conclusive evidence exists about the safety of stopping DMT after several years of stability and that observational studies have indicated negative effects of discontinuation on disability progression – suggest to consider discontinuing treatment under close clinical and MRI monitoring after 5 years of stability. Wiendl et al. [6] strongly recommend that patients who are stable on a given treatment should continue therapy as long as they do not have any safety or tolerability issues. Pending results of ongoing controlled studies assessing the risk-benefit profile of treatment discontinuation [17, 18] I endorse their reasoning in daily practice.

*Question 4* refers to the interdependence of scientific evidence and the framework provided by the official labels as defined by regulators in the respective health care system. Bayas et al. argue that the mode of action and the available evidence from phase II and observational studies document a high grade of similarity between the chimeric anti-CD20 antibody Rituximab and the humanized anti-CD20 antibody Ocrelizumab and therefore allow including Rituximab - although not approved for MS - as an equivalent option in their recommendations. Wiendl et al. propose to adhere to the official labels and to restrict off label use to those situations where approved, equally effective and tolerable compounds are not available. I concur, because regulators have a legally defined role in balancing risk-benefit of therapeutics and the interest of society including financial considerations. As treating physicians and as clinical scientists we should respect this role as long as it doesn’t compromise the best interest of our patients.

*In conclusion* there is no doubt that treatment of multiple sclerosis has radically improved the lifes and mid- to long term expectation of people diagnosed with MS of being able to conduct a normal life. But there is still enough to improve, especially regarding the better control and even more the prevention of disease progression. Great part of the current controversy is due to differing views about the affordable risk in relation to the achievable benefits of treatment with the available DMTs. Systematic comparative studies, both controlled and observational, together with the comprehensive evaluation of pwMS in all phases of their disease will hopefully produce the kind of evidence needed to better inform this debate and personalized treatment decisions.
